# Beyond Numbers: Psychological Distress and Mental Health Change Following Hybrid Closed-Loop Insulin Therapy in Adults with Complex Type 1 Diabetes

**DOI:** 10.1192/bjo.2026.11257

**Published:** 2026-06-30

**Authors:** Sunandan Banerjee, Annamarie Jones, Navya Basavaraju, Probal Moulik

**Affiliations:** 1 Black Country Healthcare NHS Foundation Trust, Birmingham, United Kingdom; 2 Shrewsbury and Telford Hospital NHS Trust, Shrewsbury, United Kingdom

## Abstract

**Aims:**

Type 1 diabetes (T1D) requires relentless self-management and carries a substantial psychological burden. In individuals with complex or disengaged diabetes, this burden frequently manifests as diabetes distress, anxiety, low mood, maladaptive coping behaviours, and disengagement from treatment. These presentations often overlap with psychiatric symptomatology yet remain under-recognised within mental health services. This study aimed to explore the lived psychological experience of adults with complex T1D and to examine changes in psychological distress following initiation of continuous subcutaneous insulin infusion (CSII) with hybrid closed-loop (HCL) technology.

**Methods:**

This work formed part of a prospective Quality Improvement Project within an NHS diabetes service. Semi-structured psychosocial questionnaires were completed by adults with T1D and significant diabetes distress prior to initiation of CSII/HCL (n=12). A subset of participants (n=7) completed the same questionnaire six months after commencing CSII/HCL. Data were analysed using inductive thematic analysis, grounded in patient narratives. The project was conducted as a service evaluation with organisational governance approval; individual consent was obtained as part of routine clinical care.

**Results:**

Pre-CSII/HCL narratives revealed a profound psychological burden. Four dominant themes emerged: (1) chronic cognitive and emotional exhaustion driven by continuous monitoring and decision-making demands; (2) anxiety-driven behaviours, including intentional hyperglycaemia to avoid hypoglycaemia, driving risk, or social embarrassment; (3) depressive cognitions characterised by hopelessness, self-blame, and perceived inevitability of complications; and (4) identity disruption, stigma, and defensive disengagement from care.

Post-CSII/HCL questionnaires demonstrated consistent qualitative change. Participants described reduced mental load, improved confidence in diabetes management, decreased fear of hypoglycaemia, and greater emotional stability. Several reported improvement in mood and anxiety symptoms, re-engagement with daily activities, and enhanced quality of life without escalation of antidepressant or anxiolytic medication. While psychological distress was not eliminated, participants described a shift from crisis-driven coping to a more manageable and contained relationship with diabetes.

**Conclusion:**

Apparent “non-compliance” in T1D often represents a defensive response to untreated psychological distress rather than behavioural failure. Hybrid closed-loop technology may function as both a metabolic and psychological intervention by reducing cognitive burden and restoring self-efficacy. These findings highlight a clear role for psychiatry in diabetes care: screening for diabetes-related distress, anxiety, and mood symptoms, supporting engagement with technology, and contributing to integrated care pathways where improving confidence in physical illness management can meaningfully improve mental health outcomes without default reliance on psychotropic medication.

